# A predictive and prognostic model for hepatocellular carcinoma with microvascular invasion based TCGA database genomics

**DOI:** 10.1186/s12885-021-09047-1

**Published:** 2021-12-16

**Authors:** Jin Wang, Zhi-Wen Ding, Kuang Chen, Yan-Zhe Liu, Nan Li, Ming-Gen Hu

**Affiliations:** 1 Faculty of Hepato-Biliary-Pancreatic Surgery, Chinese People’s Liberation Army (PLA) General Hospital, 28 Fuxing Road, Beijing, 100853 China; 2 Eastern Hepatobiliary Surgery Hospital, Second Military Medical University, 225 Changhai Road, Shanghai, 200433 China

**Keywords:** Hepatocellular carcinoma, Microvascular invasion, TCGA database, Differentially expressed genes, Prognostic value

## Abstract

**Background:**

Microvascular invasion (MVI) adversely affects postoperative long-term survival outcomes in patients with hepatocellular carcinoma (HCC). There is no study addressing genetic changes in HCC patients with MVI. We first screened differentially expressed genes (DEGs) in patients with and without MVI based on TCGA data, established a prediction model and explored the prognostic value of DEGs for HCC patients with MVI.

**Methods:**

In this paper, gene expression and clinical data of liver cancer patients were downloaded from the TCGA database. The DEG analysis was conducted using DESeq2. Using the least absolute shrinkage and selection operator, MVI-status-related genes were identified. A Kaplan-Meier survival analysis was performed using these genes. Finally, we validated two genes, HOXD9 and HOXD10, using two sets of HCC tissue microarrays from 260 patients.

**Results:**

Twenty-three MVI-status-related key genes were identified. Based on the key genes, we built a classification model using random forest and time-dependent receiver operating characteristic (ROC), which reached 0.814. Then, we performed a survival analysis and found ten genes had a significant difference in survival time. Simultaneously, using two sets of 260 patients’ HCC tissue microarrays, we validated two key genes, HOXD9 and HOXD10. Our study indicated that HOXD9 and HOXD10 were overexpressed in HCC patients with MVI compared with patients without MVI, and patients with MVI with HOXD9 and 10 overexpression had a poorer prognosis than patients with MVI with low expression of HOXD9 and 10.

**Conclusion:**

We established an accurate TCGA database-based genomics prediction model for preoperative MVI risk and studied the prognostic value of DEGs for HCC patients with MVI. These DEGs that are related to MVI warrant further study regarding the occurrence and development of MVI.

**Supplementary Information:**

The online version contains supplementary material available at 10.1186/s12885-021-09047-1.

## Introduction

Hepatocellular carcinoma (HCC) is the sixth most common malignant cancer and the third most common cause of cancer-related death worldwide [1]. With advances in surgical technology, partial hepatectomy and liver transplantation are the most commonly used elective curative treatments [2]. Unfortunately, approximately 70% of HCC patients have a recurrence within the first 5 years after R0 liver resection [3], which has seriously limited the prognosis of HCC patients. It is, therefore, necessary to find selective genetic biomarkers that can identify aggressive behaviour and predict early tumour recurrence after liver resection and transplantation.

Microvascular invasion (MVI), defined as the invasion of tumour cells in the intrahepatic portal vein or hepatic vein branches, has generally been considered as one of most vital risk factors for the overall survival (OS) and recurrence-free survival (RFS) rates of postoperative HCC patients [4–6]. Previous studies have shown the prevalence of MVI in specimens obtained from LR or transplantation to be between 15.0 and 57.1% [7]. The presence of MVI is a histopathologic feature that indicates aggressive behaviour of the HCC and predicts a worse prognosis of patients after R0 LR. Even for patients with small HCCs or those treated with transplantation or LR, MVI still increases the rate of tumour recurrence and dramatically shortens long-term survival [8].

Until now, the diagnosis of MVI has only been determined by histologic examination of the surgical specimens obtained after LR or transplantation [7, 9, 10]. The influence of the diagnosis on preoperative decision making is limited. An accurate preoperative estimation of MVI presence can help hepatic surgeons choose appropriate surgical procedures for patients based on risk-benefit assessment [11]. Hence, many efforts to obtain a preoperative estimation of MVI have been made over the past decade [7]. Recent studies reported that imaging may play an important role in preoperative prediction of MVI [12–14]. Based on some radiomics signatures, a radiomics nomogram showed a favourable predictive accuracy for MVI status in patients with HBV-related HCC [15]. The use of serum or tumour biomarkers to estimate MVI risk has also been proposed [16, 17]. Unfortunately, these serum markers can also be abnormally high in patients with benign liver disease. Another predictive model that incorporates factors associated with MVI based on preoperative clinicopathologic data was reported [11]. However, all the available models failed to examine the patients’ genetic changes related to the occurrence and progression of MVI. Owing to this lack of a specific and practical genomics predictive method, the development of a predictive model that incorporates genetic changes associated with MVI becomes desirable. Additionally, it is vital to screen genes associated with MVI and confirm their prognostic value for exploring the deep mechanism of occurrence and progression of MVI.

To our knowledge, we have established the first genomics prediction model, based on the TCGA database and patients’ sample validation, for preoperative MVI risk estimation in HCC. In addition, we first screen out genetic changes associated with MVI and confirmed the prognostic value of these for HCC patients with MVI. Further study concerning the occurrence and development of MVI is warranted.

## Materials and methods

### Data collection and pre-processing

The mRNA raw count profiles of HCC patients were downloaded from TCGA dataset (https://tcga-data.nci.nih.gov/tcga/). A total of 374 HCC patients’ samples and 50 control samples were available in TCGA, and the full clinical dataset also was downloaded. Simultaneously, the count data were normalized using the VST algorithm implemented in DEseq2 package.

### DEG_T-N_ identification

**Fig. 1:**
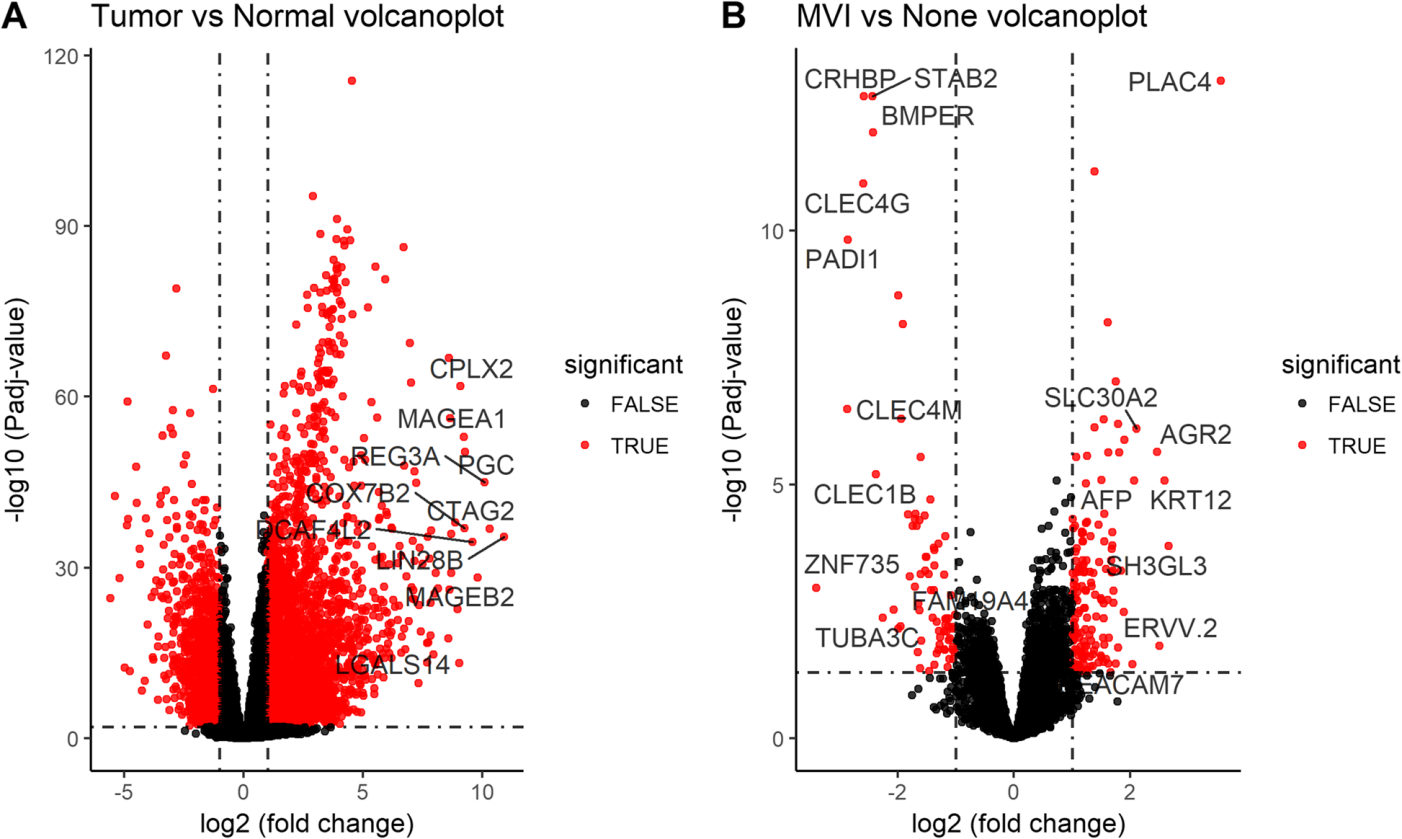
The volcano plots of differentially expressed mRNAs. The plots were constructed using fold-change values and Padj values. The vertical lines correspond to 2.0-fold changes up and down and the horizontal line represents a Padj values. The red dots in the plot represent the differentially expressed mRNAs with their level of statistical significance. **A** Tumour samples and non-tumour samples in hepatocellular carcinoma. **B** MVI samples and non-MVI samples in hepatocellular carcinoma

The differentially expressed genes (DEG_T-N_) in HCC samples and control tissues were identified using R package “DESeq2” with a cut-off of |log2-fold change| > 1 and Padj < 0.01 (*P*-value adjusted for multiple testing using Benjamini-Hochberg method) (see Fig. 1A).

### DEG_M-N_ identification

Among 374 patients’ samples, after excluding the samples with missing vascular invasion status or macroscopic vascular invasion, 299 samples were included in this study for next analysis, consisting of 93 MVI samples and 206 non-vascular invasion. Among DEG_T-N_, the differentially expressed genes (DEG_M-N_) in the MVI samples and non-vascular invasion samples were identified using “DESeq2” R package with a cut-off of |log2-fold change| > 1 and Padj < 0.05.

### Selection of MVI-status-related key genes

A logistical model was built using the least absolute shrinkage and selection operator (LASSO) algorithm. To find an optimal λ, 10-fold cross validation with minimum criteria was employed, and the λ with the smallest cross-validation error was chosen. Other parameters were set to default values. Finally, 23 key genes were identified, and the LASSO logistical regression modelling was performed using R package “glmnet”.

### Construction and validation of the classification model

In machine-learning, the classification is considered as a supervised learning task of inferring a function from labelled training data. Random forest is considered as an appropriate classifier to handle large-scale dataset, especially for imbalanced dataset, because of its ability to provide an empirical approach to monitor variable interactions. In this study, we randomly divided the 299 samples into training datasets (*N* = 225) and test datasets (*N* = 74). Based on 23 MVI-status-related genes, random forest was adopted to construct the classification model in the training datasets, and a classification model was implemented using R package “caret”. The classifier was trained using the repeated (10 repeat iterations) 10-fold cross validation of training datasets, and their predictive performance was evaluated in the test datasets using area under ROC curve (AUC).

### One-way ANOVA and survival analysis

We adopted one-way ANOVA to demonstrate the association between the MVI-status-related genes and the corresponding clinical features, and the statistical significance was shown by boxplot. Then, to evaluate the impact of MVI-status-related genes on MVI-HCC patient’s prognosis, the overall survival was analysed. Among 93 MVI-HCC patients, survival time was missing for one sample. We performed a survival analysis for the remaining 92 samples. Continuous variables were dichotomized for OS before the log-rank test using optimal cut-off values determined by the “surv_cutpoint” function of the “survminer” R package. We adopted “survival” package of R software for log-rank test and Kaplan-Meier survival analysis.

### HCC tissue samples

Two hundred and sixty pairs of primary HCC tissues and their corresponding adjacent normal tissues were obtained from patients who underwent hepatectomy between February, 2002 and June 2006 at the Eastern Hepatobiliary Surgery Hospital, Second Military Medical University, Shanghai, China. All of the patients signed an informed consent. All of the patients met the following inclusion criteria and underwent tissue microarrays (TMA) analysis: (a) a definite clinical diagnosis and postoperative pathological diagnosis of HCC, (b) R0 resection of all patients based on histologic examinations, (c) complete clinicopathologic and follow-up data, (d) absence of distant metastasis, and (e) absence of treatment before surgery. Our study was approved by the Institutional Review Board of the Institute for Eastern Hepatobiliary Surgery Hospital, Second Military Medical University.

### TMAs and IHC

The tissue microarray slides were stained using the semi-quantitative system according to the manufacturer’s instructions, with a rabbit polyclonal antibody (Abcam, ab90260, 1:100, Abcam, ab85698, 1:100). Multispectral images (8 bit) acquired by the Vectra platform (Perkin-Elmer, Waltham, MA) were processed with Nuance 30.0 software (Perkin-Elmer, Waltham, MA) to build unique spectral curves for each of the four chromogens, and then unmix the signals of multispectral images. Staining intensity as a measure of selected candidate gene expression was quantified by the optical density of the respective chromogen per unit area in pixels. Cells positive for selected candidate gene were counted by colocalization analysis using Inform TM 2.1 software. The following tissue cores were excluded from analysis: a) less than 5% epithelial component, b) significant tissue loss or folds, and c) images with more than 5% poorly segmented nuclei. The histological-score (H-score) was used to evaluate the staining intensity. For each observed tissue component, a summary value we refer to as H-Score was calculated.

### Follow-up

All patients were re-examined for serum AFP levels, liver function, and abdominal USG once a month. Chest X-rays and contrast CT or MRI scan were performed every 2–3 months for monitoring recurrence. The overall survival (OS) was defined as the interval between surgery and the date the patient died. Recurrence-free survival (RFS) was defined as the interval between surgery and the date of recurrence [18]. The OS and RFS were censored at the last follow-up visit (August 31, 2012) for surviving patients and those without recurrence. Micro-metastases were defined as tumours adjacent to the border of the main tumour that could only be observed under a microscope.

### Statistical analysis

All statistical analyses were performed using SPSS 22.0 statistical software. A chi-square test was used to evaluate the association between the selected candidate gene expression level and the clinicopathological characteristics. We used the Kaplan-Meier method to evaluate the cumulative survival, and the significance of the differences was determined using the log-rank test. Furthermore, we used Cox multivariate regression analysis to determine the independent prognostic factors of HCC. The statistical results were considered significant at *P* < 0.05 and highly significant at *P* < 0.01.

## Results

### Identification of DEG_T-N_ and DEG_M-N_

A total of 4357 DEG_T-N_ (3155 upregulated and 1203 downregulated genes) were identified for subsequent analysis at the threshold of |log2-fold change| > 1 and Padj < 0.01(see Fig. 1A). Overall, 223 differentially expressed genes (DEG_M-N_) between MVI samples and non-vascular invasion samples were identified from 4357 differentially expressed genes (DEG_T-N_) in the HCC samples and non-cancerous tissues (see Fig. 1B).

### Identification of MVI-status-related key genes

**Fig. 2:**
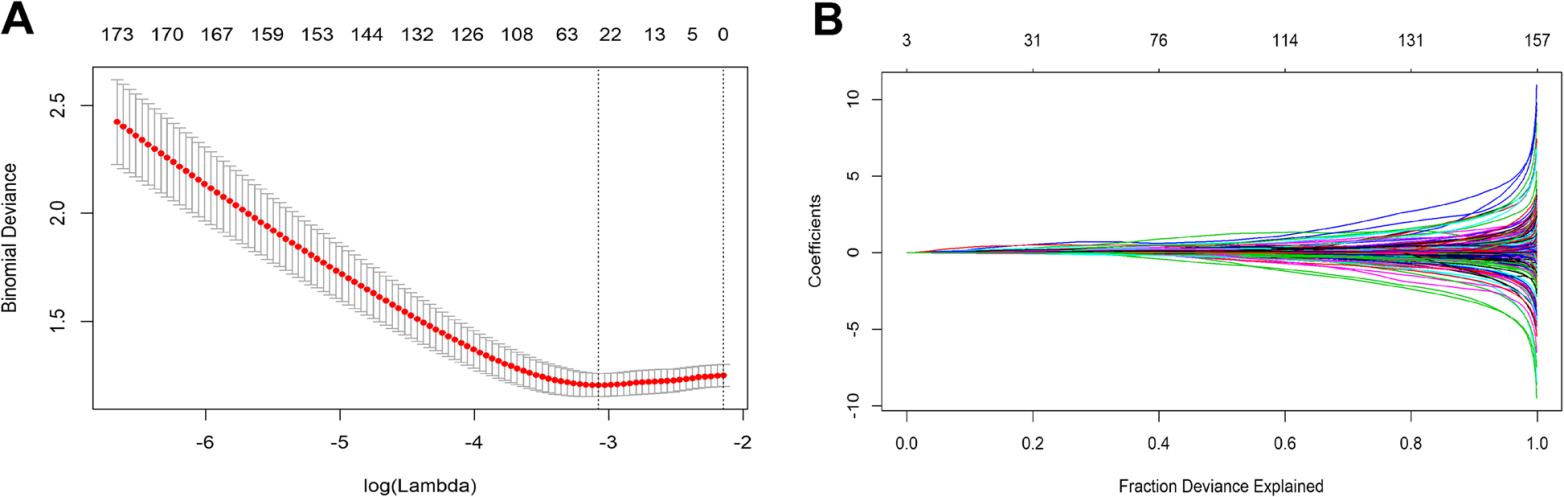
Selection of optimal tuning parameter *λ* in the LASSO model. **A** The dotted vertical lines are drawn at the optimal values according to minimum criteria (left) and 1-SE criteria (right). The optimal *λ* = 0.0461 was determined by 10-time cross-validation via minimum criteria. Error bars represent the standard error (SE). **B** LASSO coefficient profiles of the 223 associated mRNAs. A vertical line is drawn at the value determined by the 10-fold cross validation

We performed LASSO logistical regression to identify genes associated with MVI-status using DEG_M-N_. At the optimal λ = 0.0461 in LASSO logistical regression model, the 10-fold cross validation error was minimized (see Fig. 2A). LASSO coefficient profiles of the genes are shown in Fig. 2B. Finally, 23 key genes were identified based on their non-zero regression coefficients. Through screening the TCGA database, transporting subunit alpha 4 (ATP1A4), cystatin like 1 (CSTL1), forkhead box D3 (FOXD3), hypocretin neuropeptide precursor (HCRT), homeobox D10 (HOXD10), keratin 12 (KRT12), protocadherin alpha 1 (PCDHA1), scavenger receptor class A member 5 (SCARA5), solute carrier family 25 member 47 (SLC25A47), very low density lipoprotein receptor (VLDLR), adrenoceptor alpha 1D (ADRA1D), aldehyde dehydrogenase 3 family member A1 (ALDH3A1), chromosome 1 open reading frame 61 (C1orf61), calcium voltage-gated channel auxiliary subunit gamma 4 (CACNG4), CUGBP elav-like family member 5 (CELF5), homeobox D9 (HOXD9), keratin associated protein 5–7 (KRTAP5–7), nuclear pore complex interacting protein family member B13 (NPIPB13), opiorphin prepropeptide (OPRPN), olfactory receptor family 1 subfamily F member 1 (OR1F1), proprotein convertase subtilisin/kexin type 1 inhibitor (PCSK1N), thyroid hormone responsive (THRSP), and transmembrane serine protease 15 (TMPRSS15) were identified in HCC with non-MVI group and HCC with MVI group.

### Building a classification model and validation

**Fig. 3:**
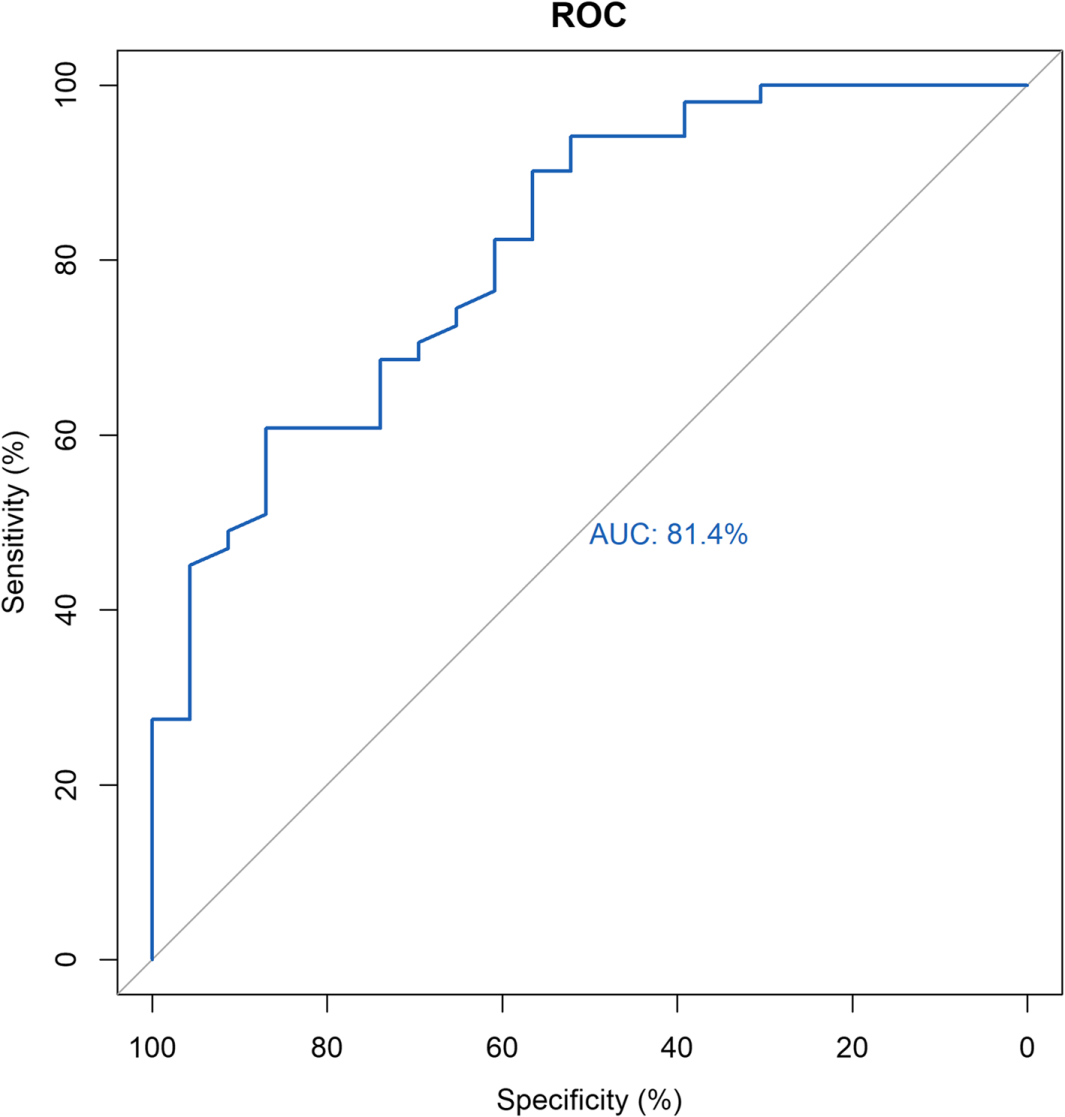
ROC analysis of the classification model applied to the TCGA database. Receiver operating characteristic (ROC) curves and area under the curve (AUC) statistics evaluate the capacity of distinguishing MVI and non-MVI

In this study, 299 samples were randomly divided into training datasets (*N* = 225) and test datasets (*N* = 74). Based on 23 MVI-status-related key genes, we performed a Random Forest machine learning approach to construct a classification model using training datasets. The test datasets were used to validate the predictive performance. The area under receiver operator characteristic curve (AUC) was used to assess the predictive performance of classification model. In our study, the area under curve (AUC) of random forest (RF) classification model reached 0.814 (see Fig. 3), demonstrating its good performance for classification.

### One-way ANOVA and survival analysis

**Fig. 4:**
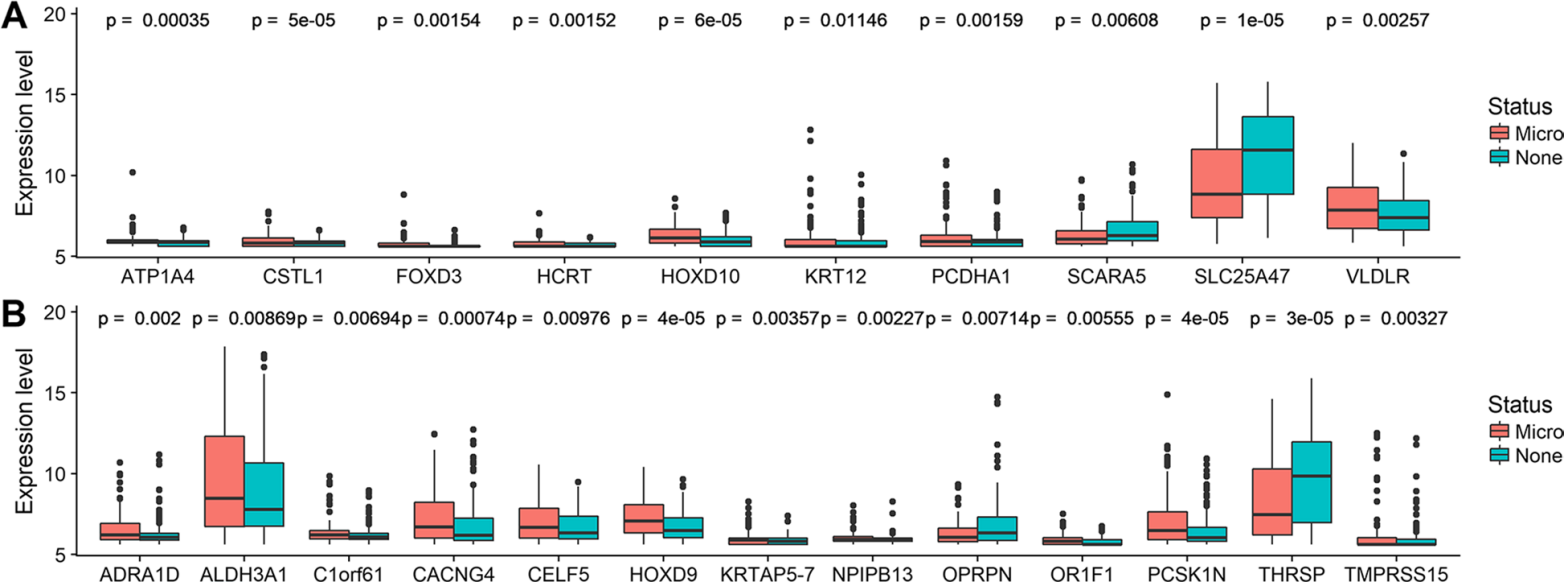
The boxplots show the medians and dispersions between MVI samples and non-MVI samples for each key gene. **A** Boxplots of hub genes from ATP1A4 to VLDLR (in alphabetical order) in different invasion statuses. **B** Boxplots of hub genes from ADRA1D to TMPRSS15 in different invasion statuses. *P*-values are the results of one-way ANOVA for different invasion statuses. Micro means MVI sample. None means non-MVI samples

**Fig. 5:**
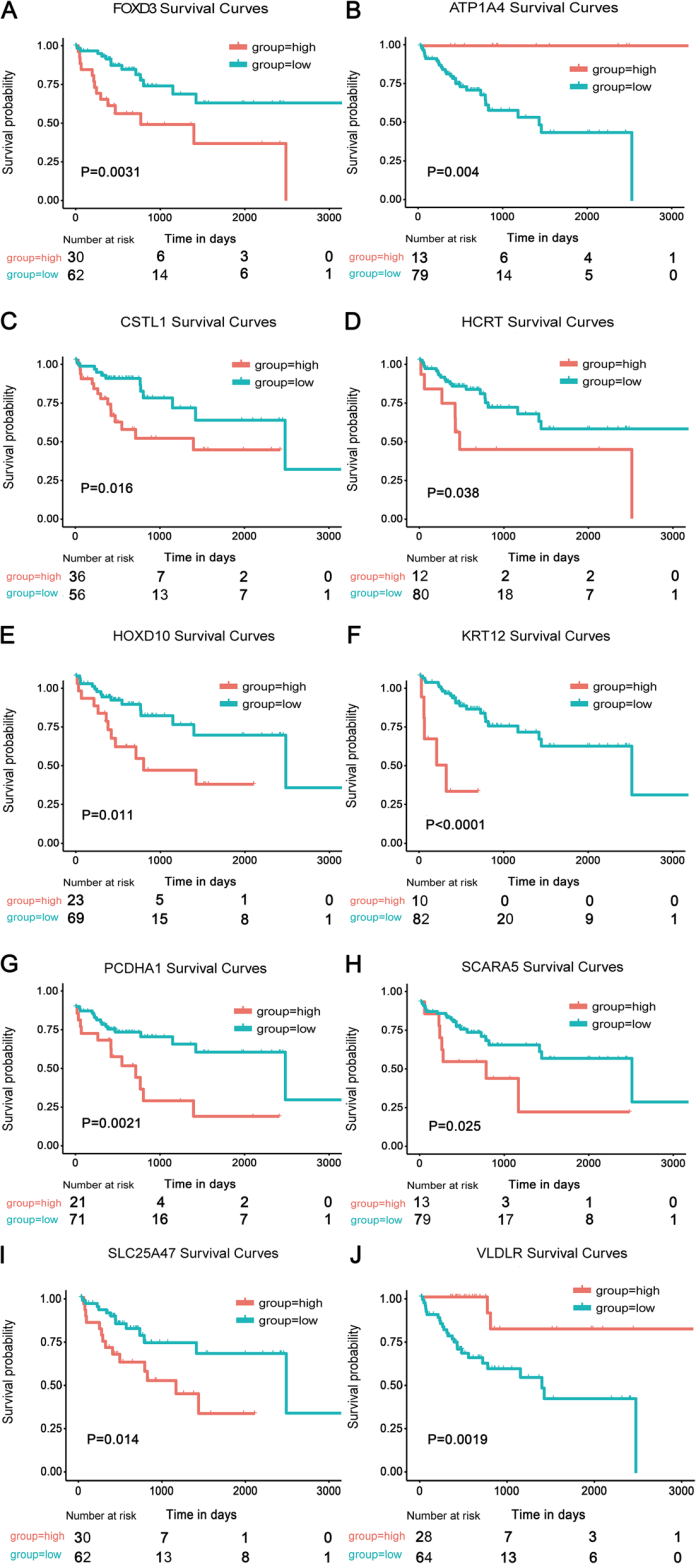
Survival analysis of the ten key genes in the TCGA dataset. **A** FOXD3. **B** ATP1A4. **C** CSTL1. **D** HCRT. **E** HOXD10. **F** KRT12. **G** PCDHA1. **H** SCARA5. **I** SLC25A47. **J** VLDLR. Red lines represent high expression of the key genes, and blue lines represent low expression

In this study, first, we calculated the statistical significance of the difference between the MVI-status and non-MVI-status. The result showed the expression level of 23 key genes had a significant difference (see Fig. 4). Additionally, the 92 MVI-HCC patients were divided into low-level group and high-level group according to optimal cut-off values of each hub gene, and the survival curve was plotted. We found the survival time of low level group was significantly longer than the high level group (see Fig. 5), indicating that nearly half of the key genes could act as prognosis biomarkers of MVI-HCC patients. The survival curve of the remaining thirteen genes was also plotted (see supplementary Fig. 1).

### Validation group of two sets of HCC tissue microarrays

**Fig. 6:**
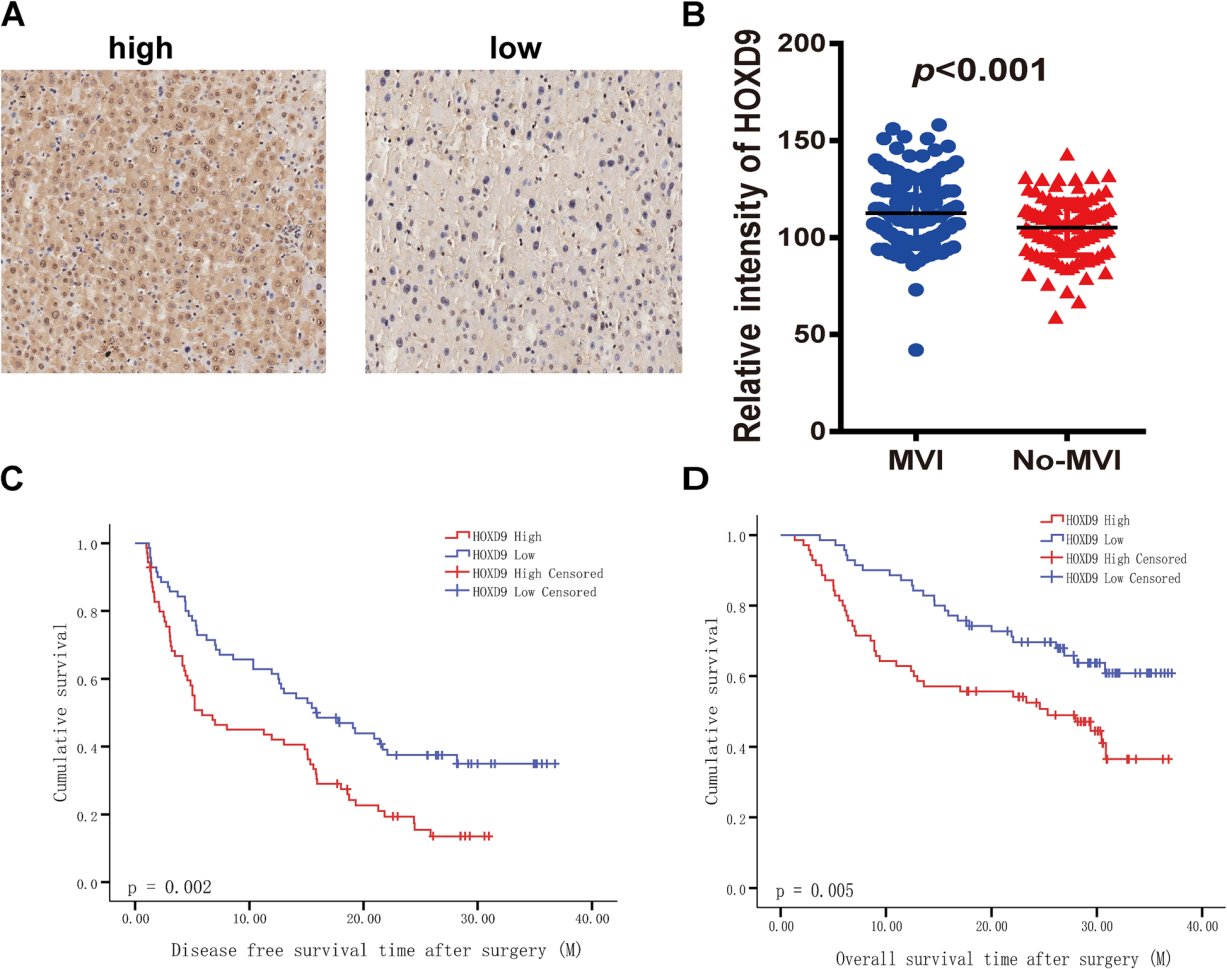
Validation group of HOXD9 in one set of HCC tissue microarrays. **A** Immunohistochemistry staining of HOXD9 in HCC tissues with MVI and without MVI. **B** Scatter plots reflecting the HOXD9 staining intensity in HCC tissues with MVI (*n* = 140) and without MVI (*n* = 120); ***, *P* < 0.001. **C** High HOXD9 expression correlated with lower disease-free survival in HCC patients (HOXD9 high, *n* = 70; HOXD9 low, *n* = 70), *P* = 0.002. **D** High HODX9 expression correlated with lower overall survival in HCC patients (HOXD9 high, *n* = 70; HOXD9 low, *n* = 70), *P* = 0.005

**Fig. 7:**
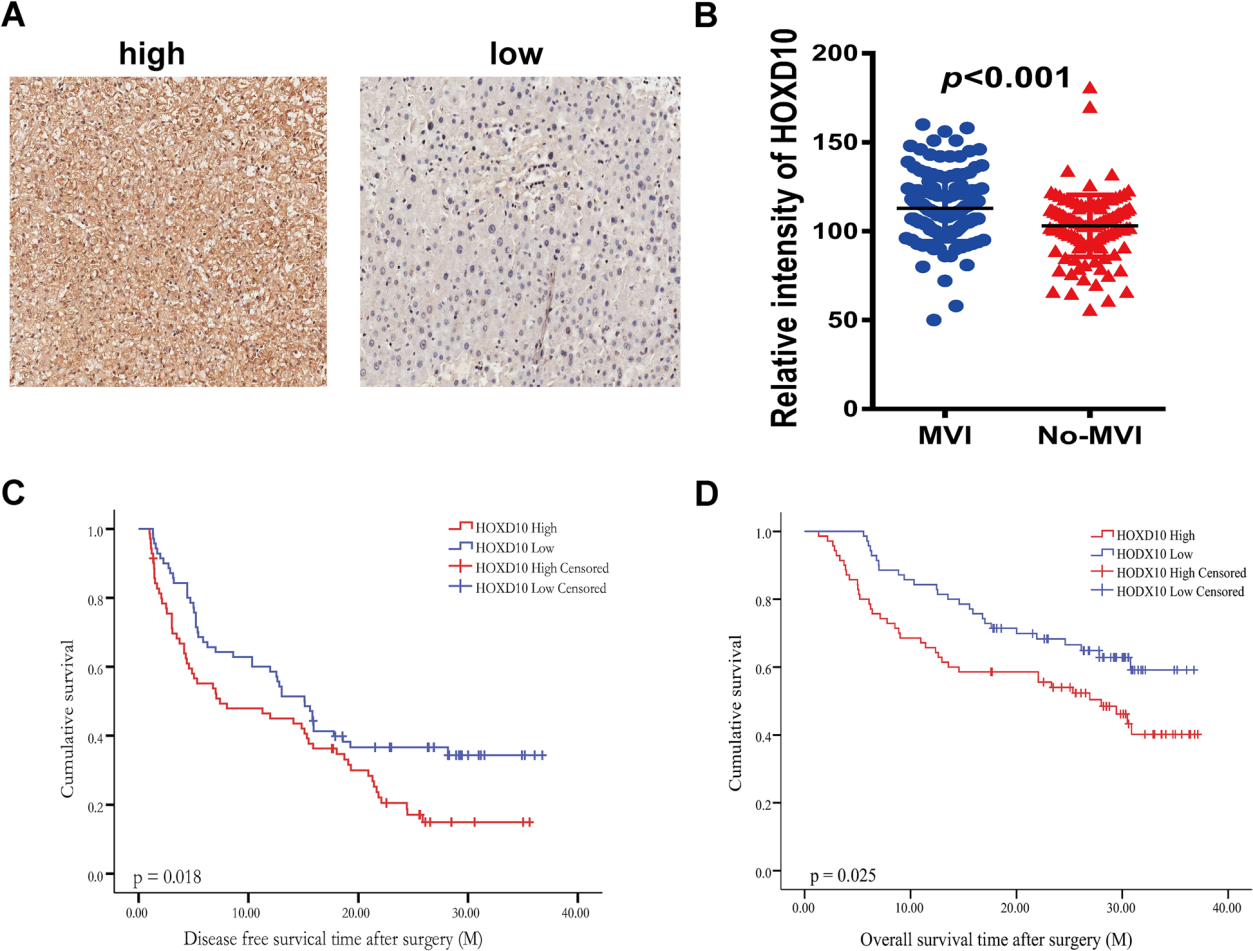
Validation group of HOXD10 in one set of HCC tissue microarrays. **A** Immunohistochemistry staining of HOXD10 in the HCC tissues with MVI and without MVI; **B** Scatter plots reflecting the HOXD10 staining intensity in HCC tissues with MVI (*n* = 140) and without MVI (*n* = 120); ***, *P* < 0.001. **C** High HOXD10 expression correlated with lower disease-free survival in HCC patients (HOXD10 high, *n* = 70; HOXD10 low, *n* = 70), *P* = 0.018. **D** High HODX10 expression correlated with lower overall survival in HCC patients (HOXD10 high, *n* = 70; HOXD10 low, *n* = 70), *P* = 0.025

From screening the TCGA-database, we distinguished 23 differential genes in HCC patients with non-MVI group and HCC patients with MVI group. We validated two differentially expressed genes, HOXD9 and HOXD10, and explored their prognostic value for HCC patients with MVI after liver resection, using two sets of 260 patients’ HCC tissue microarrays from XXX. To further confirm the expression of HOXD9 and HOXD10, we examined HOXD9 and HOXD10 expression in an HCC tissue microarray by immunohistochemical (IHC) staining. Based on the results of the HCC tissue microarray, HOXD9 and HOXD10 expression was higher in the HCC tissues with MVI (*n* = 140) than in HCC tissues without MVI (*n* = 120) (*P* < 0.001) (Figs. 6A, B and 7A, B).

The staining intensity of HOXD9 and HOXD10 expression was scored automatically with a Vectra2 system (Perkin Elmer, USA). According to the intensity score, the HCC patients with MVI were divided into two groups: HOXD9 and HOXD10 low (*n* = 70) and HOXD9 and HOXD10 high (*n* = 70). We explored the association between HOXD9 and HOXD10 expression and the clinicopathological features in 140 HCC patients. Moreover, we studied the association between the prognosis of HCC patients with MVI and the expression of HOXD9 and HOXD10. In Fig. 6C, D, we found that high HOXD9 high expression levels were associated with poor overall survival (OS) and recurrence-free survival (RFS) in 140 HCC patients with MVI. Similar results were obtained for HOXD10 (Fig. 7C, D). These results showed that HOXD9 and HOXD10 were upregulated in HCC tissues and that HOXD9 and HOXD10 overexpression affected the prognosis of HCC patients.

## Discussion

Microvascular invasion (MVI), also known as microvascular tumour thrombus, has been repeatedly and definitively confirmed as a poor prognostic factor of HCC tumour recurrence after R0 liver resection [9]. Preoperative prediction of MVI and exploration of the genetic characteristics of HCC patients with MVI carry important clinical significance. However, most recent studies have examined the serological and imaging features of HCC with MVI and preoperative prediction of MVI [11, 19, 20]. In addition, the genomics characteristics of HCC patients with MVI and MVI related with oncogenes and suppressor genes are unknown.

In our study, we first screened for genes associated with MVI that were different from normal liver tissue and HCC without MVI based on the TCGA database and studied the prognostic value of these in HCC patients with MVI. We established a TCGA database-based genomics prediction model for MVI. These selected genes related with MVI warrant further study concerning the occurrence and development of MVI.

In previous studies, MVI was only identifiable under a microscope based on postoperative pathological diagnosis. Its presence has been confirmed as a poor prognostic factor of HCC recurrence and of the survival outcomes of HCC patients.

The influence of the diagnosis on preoperative decision making is limited. If liver resection is considered for patients with a high risk of MVI, then a wide resection margin might be the preferred procedure with curative intent [5, 6, 21, 22]. Recent studies have mainly focused on the imaging and serological characteristics of HCC patients with MVI and ignored the genetic changes in these patients. A radiomics nomogram for preoperative prediction of MVI risk in hepatitis B virus-related HCC was reported [15]. This nomogram integrated radiomics signature with radiologic features and AFP level to further improve its predictive accuracy for MVI. Another nomogram for preoperative MVI risk estimation in HCC based on preoperative clinicopathologic data was also described [11]. However, recent studies related with MVI have important limitations. The genetic differences among normal tissue, HCC without MVI tissue, and HCC with MVI tissue remain unknown. Based on the above reasons, basic research about the genetic characteristics of occurrence and progression of MVI has been limited. We screened for genes from TCGA databases with distinct differences between HCC with or without MVI to search for these genes that may play an important role in the occurrence and development of MVI. Furthermore, we established TCGA database-based genomics prediction model of MVI and explored the prognostic value of 23 genes related to MVI for HCC with MVI, validated with two tissue chips of 260 patients.

In recent studies, a nomogram for preoperative estimation of MVI risk in hepatitis B virus-related HCC within the Milan criteria was established based on 7 factors: large tumour diameter, multiple nodules, incomplete capsule, α-fetoprotein level greater than 20 ng/mL, platelet count less than 100 × 10^3^/μL, hepatitis B virus DNA load greater than 10^4^ IU/mL, and a typical dynamic pattern of tumours on contrast-enhanced magnetic resonance imaging [11]. This nomogram achieved concordance indexes of 0.81 (95% CI 0.78–0.85) and 0.80 (95% CI 0.75–0.86) in predicting MVI in the training and validation cohorts, respectively. Another radiomics nomogram for preoperative prediction of microvascular invasion risk in hepatitis B virus-related HCC, including the radiomics signature, nonsmooth tumour margin, hypoattenuating halos, internal arteries, and alpha-fetoprotein level, showed a favourable predictive accuracy for MVI status in patients with HBV-related HCC [15]. However, our study examined the genomics changes of HCC patients with MVI, integrating 23 differentially expressed genes (DEGs) between HCC patients with MVI and without MVI, to predict the preoperative MVI risk with an AUC of 81.4%. This TCGA database-based genomics prediction model was more accurate in predicting the MVI risk than that of serological and imaging indicators.

No studies have examined the genetic changes for HCC patients with MVI. In our study, we first screened 23 DEGs in HCC patients with MVI and without MVI. These DEGs may be associated with the occurrence and development of MVI, and the genomic characteristics of MVI. Furthermore, we explored the prognostic value of 23 DEGs for HCC patients with MVI, validated by HCC tissue microarrays of 260 patients. Among DEGs, we selected homeobox (HOX) genes 9 and 10 to validate the expression and prognostic value of our screened DEGs. HOX genes encode a highly conserved family of transcription factors that significantly influence many cellular processes, including proliferation, apoptosis, cell shape, and cell migration. HOX genes contain a conserved 183 bp sequence and encode nuclear proteins called homeoproteins [23–25]. In neoplasms such as laryngeal squamous cells, HOX proteins participate in proliferation and oncogenic transformation [26, 27]. Studies have demonstrated the relation of HOXD9 to epigenetic control in development and diseases [28]. Our study indicated that HOXD9 and 10 were overexpressed in HCC patients with MVI compared with patients without MVI, and patients with MVI with HOXD9 and 10 overexpression had poorer prognosis than patients with MVI with low expression of HOXD9 and 10. Similar results were obtained in previous studies. Lv et al. reported that HOXD9 promotes epithelial–mesenchymal transition and cancer metastasis by regulation of ZEB1 in HCC [29]. In addition, MVI was regarded as an early biomarker of metastasis of HCC, indicating that HOXD 9 was related with the occurrence and development of HCC with MVI. Therefore, we screened 23 DEGs associated with MVI and identified genetics changes in HCC patients with MVI. In addition, the related genes of tumor microenvironment and presence of MVI should be further explored [30, 31].

This study has some limitations. First, we screened DEGs based on the TCGA database, which contains incomplete information about DEGs related with HCC patients with MVI. Second, we validated the prognostic value of two DEGs for HCC with MVI. However, all 23 DEGs should be incorporated in predicting the prognostic value of HCC patients with MVI. Finally, all 23 differentially expressed genes (DEGs) in HCC patients with MVI and without MVI for predicting the preoperative MVI risk need to be further validated in clinical practice.

## Conclusions

In conclusion, we first screened for DEGs associated with HCC patients with MVI that were different from normal and HCC patients without MVI based on the TCGA database and studied and validated the prognostic value of these in HCC patients with MVI. We established a TCGA database-based genomics prediction model for accurately assessing the preoperative MVI risk. These DEGs related to MVI warrant further study of the occurrence and development of MVI.

## Supplementary Information

**Additional file 1.**

## Data Availability

Availability of data and materials from TCGA dataset (https://tcga-data.nci.nih.gov/tcga/).

## Abbreviations

HCC: Hepatocellular carcinoma
MVI: Microvascular invasion
OS: Overall survival
RFS: Recurrence-free survival
LR: Liver resection
DEGs: Differentially expressed genes
TMA: Tissue microarrays
ROC: Receiver operator characteristic curve
AUC: Area under curve
RF: Random forest
