# Supplementary material for: A predictive and prognostic model for hepatocellular carcinoma with microvascular invasion based TCGA database genomics
Source: BMC Cancer. 2021 Dec 16;21:1337. doi: 10.1186/s12885-021-09047-1 (PMC8675478; doi:10.1186/s12885-021-09047-1)
Supplement: Supplementary file 1 — Additional file 1. [file 12885_2021_9047_MOESM1_ESM.doc]

**TCGA database-based genomics predictive and prognostic model for hepatocellular carcinoma with microvascular invasion**

**Authors:**

Jin Wang^1^*, Zhi-Wen Ding^2^*, Kuang Chen^1^, Yan-Zhe Liu^1^, Nan Li^2#^, Ming-Gen Hu^1#^

**Affiliations:**

1. Faculty of Hepato-Biliary-Pancreatic Surgery, Chinese People’s Liberation Army (PLA) General Hospital, Beijing, China.

2. Eastern Hepatobiliary Surgery Hospital, Second Military Medical University, Shanghai, China.

***These authors contributed equally to this work.**

**Corresponding authors:** **Ming-Gen Hu,** M.D. Faculty of Hepato-Biliary-Pancreatic Surgery, Chinese People’s Liberation Army (PLA) General Hospital, 28 Fuxing Road, Beijing 100853, China, E-mail: hmg301@126.com. OR **Nan Li**, M.D., Eastern Hepatobiliary Surgery Hospital, Second Military Medical University, 225 Changhai Road, Shanghai, 200433 China, E-mail: liparislisi@aliyun.com

**Supplementary Table 1. Clinicopathological Features of 140 HCC Specimens with MVI in Validation group of HOXD9 in one set of HCC tissue microarrays.**

| Characteristics |  | HOXD9 low | HOXD9 high | p value |
| --- | --- | --- | --- | --- |
| Age(year) | ≤50 | 35(44.87%) | 35(56.45%) | 0.173 |
|  | >50 | 43(55.13%) | 27(43.55%) |  |
| Gender | Male | 62(49.21%) | 8(57.14%) | 0.573 |
|  | Female | 64(50.79%) | 6(42.86%) |  |
| HBsAg | Positive | 3(30.00%) | 67(51.54%) | 0.189 |
|  | Negetive | 7(70.00%) | 63(48.46%) |  |
| AFP(μg/L) | ≤400 | 17(38.64%) | 53(55.21%) | 0.069 |
|  | >400 | 27(61.36%) | 43(44.79%) |  |
| AST(μg/L) | ≤40 | 37(49.33%) | 33(50.77%) | 0.865 |
|  | >40 | 38(50.67%) | 32(49.23%) |  |
| Tumor size(cm) | ≤5 | 17(50.00%) | 43(50.00%) | 1.000 |
|  | >5 | 17(50.00%) | 43(50.00%) |  |
| Tumor number | Single | 64(50.79%) | 6(42.86%) | 0.573 |
|  | Multiple | 62(49.21%) | 8(57.14%) |  |
| Hepatocirrhosis | Yes | 43(51.19%) | 27(48.21%) | 0.730 |
|  | No | 41(48.81%) | 29(51.79%) |  |
|  |  |  |  |  |

HBsAg, hepatitis B virus surface antigen; AFP, α-fetoprotein; AST, Aspartate aminotransferase

**Supplementary Table 2. Clinicopathological Features of 140 HCC Specimens with MVI in Validation group of HOXD10 in one set of HCC tissue microarrays.**

| Characteristics |  | HOXD10 low | HOXD10 high | p value |
| --- | --- | --- | --- | --- |
| Age(year) | ≤50 | 38(48.72%) | 32(51.61%) | 0.734 |
|  | >50 | 40(51.28%) | 30(48.39%) |  |
| Gender | Male | 65(51.59%) | 5(35.71%) | 0.260 |
|  | Female | 61(48.41%) | 9(64.29%) |  |
| HBsAg | Positive | 5(50.00%) | 65(50.00%) | 1.000 |
|  | Negetive | 5(50.00%) | 65(50.00%) |  |
| AFP(μg/L) | ≤400 | 24(54.55%) | 46(47.92%) | 0.466 |
|  | >400 | 20(45.45%) | 50(52.08%) |  |
| AST(μg/L) | ≤40 | 34(45.33%) | 36(55.38%) | 0.236 |
|  | >40 | 41(54.67%) | 29(44.62%) |  |
| Tumor size(cm) | ≤5 | 17(50.00%) | 43(50.00%) | 1.000 |
|  | >5 | 17(50.00%) | 43(50.00%) |  |
| Tumor number | Single | 64(50.79%) | 6(42.86%) | 0.573 |
|  | Multiple | 62(49.21%) | 8(57.14%) |  |
| Hepatocirrhosis | Yes | 45(53.57%) | 25(44.64%) | 0.301 |
|  | No | 39(46.43%) | 31(55.36%) |  |
|  |  |  |  |  |

HBsAg, hepatitis B virus surface antigen; AFP, α-fetoprotein; AST, Aspartate aminotransferase


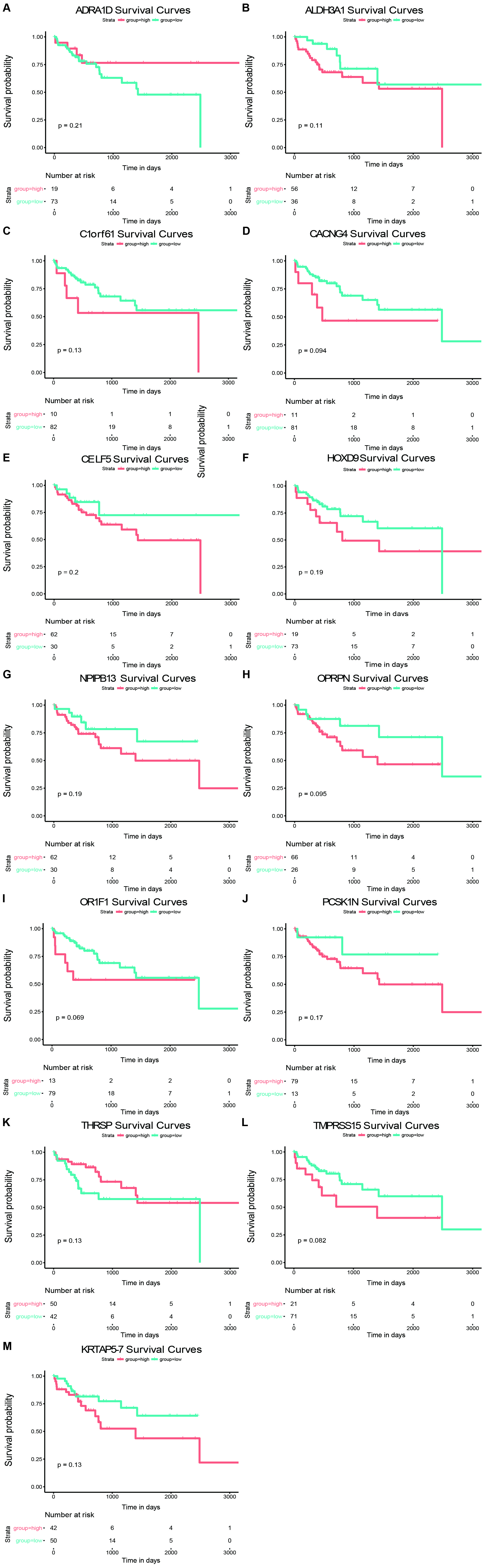


**Figure S1. Survival analysis of the thirteen key genes in the TCGA dataset.** (A) ADRA1D. (B) ALDH3A1. (C) C1orf61. (D)CACNG4. (E)CELF5. (F)HOXD9. (G) NPIPB13. (H) OPRPN. (I)OR1F1. (J)PCSK1N. (K)THRSP. (L)TMPRSS15. (M)KRTAP5-7. Red lines represent high expression of the key genes, and blue lines represent low expression.
